# Multi-Method Characterization of the Human Circulating Microbiome

**DOI:** 10.3389/fmicb.2018.03266

**Published:** 2019-01-17

**Authors:** Emma Whittle, Martin O. Leonard, Rebecca Harrison, Timothy W. Gant, Daniel Paul Tonge

**Affiliations:** ^1^School of Life Sciences, Faculty of Natural Sciences, Keele University, Keele, United Kingdom; ^2^Centre for Radiation, Chemical and Environmental Hazards, Public Health England, Chilton, United Kingdom

**Keywords:** blood microbiome, unmapped reads, human, next gen sequencing (NGS), biomarker (development)

## Abstract

The term microbiome describes the genetic material encoding the various microbial populations that inhabit our body. Whilst colonization of various body niches (e.g., the gut) by dynamic communities of microorganisms is now universally accepted, the existence of microbial populations in other “classically sterile” locations, including the blood, is a relatively new concept. The presence of bacteria-specific DNA in the blood has been reported in the literature for some time, yet the true origin of this is still the subject of much deliberation. The aim of this study was to investigate the phenomenon of a “blood microbiome” by providing a comprehensive description of bacterially derived nucleic acids using a range of complementary molecular and classical microbiological techniques. For this purpose we utilized a set of plasma samples from healthy subjects (*n* = 5) and asthmatic subjects (*n* = 5). DNA-level analyses involved the amplification and sequencing of the 16S rRNA gene. RNA-level analyses were based upon the *de novo* assembly of unmapped mRNA reads and subsequent taxonomic identification. Molecular studies were complemented by viability data from classical aerobic and anaerobic microbial culture experiments. At the phylum level, the blood microbiome was predominated by *Proteobacteria*, *Actinobacteria*, *Firmicutes*, and *Bacteroidetes*. The key phyla detected were consistent irrespective of molecular method (DNA vs. RNA), and consistent with the results of other published studies. *In silico* comparison of our data with that of the Human Microbiome Project revealed that members of the blood microbiome were most likely to have originated from the oral or skin communities. To our surprise, aerobic and anaerobic cultures were positive in eight of out the ten donor samples investigated, and we reflect upon their source. Our data provide further evidence of a core blood microbiome, and provide insight into the potential source of the bacterial DNA/RNA detected in the blood. Further, data reveal the importance of robust experimental procedures, and identify areas for future consideration.

## Background

The term “microbiome” describes the genetic material encoding the various microbial populations that inhabit our body. In contrast, the term “microbiota” refers to the viable organisms that comprise these communities. The microbiome undertakes essential biological processes and thus it is unsurprising that a number of disease states are associated with changes in microbiome composition, termed “dysbiosis.” Whilst the colonization of specific body sites in contact with the external environment (such as the gastrointestinal tract, skin, and vagina) by microorganisms is both well-described and universally accepted (Markova, 2017), the existence of microbial populations in other “classically sterile” locations, including the blood, is a relatively new concept.

The presence of bacteria-specific DNA in the blood has been reported in the literature for some time, yet the true origin of this is still the subject of much deliberation. Mounting evidence supports the existence of a blood microbiome (*specifically, the presence of bacterial genetic material*) in humans (Nikkari et al., 2001; Amar et al., 2013; Rajendhran et al., 2013; Dinakaran et al., 2014; Kell and Pretorius, 2015; Potgieter et al., 2015; Mangul et al., 2016; Païssé et al., 2016; Bhattacharyya et al., 2017; Li et al., 2018) and various other species, including rodents, cats, chickens, and cows (Sze et al., 2014; Mandal et al., 2016; Jeon et al., 2017; Vientós-Plotts et al., 2017). This has primarily been determined by amplification and sequencing of the bacterial 16S rRNA gene, or via whole genome sequencing. Such studies report the existence of bacteria-derived genetic material (DNA) within the circulation, but do not provide evidence for the presence of viable organisms.

Convention tells us that the blood is sterile in health, and bacteraemia, even at 1–10 bacterial cells per milliliter whole blood, is potentially life threatening. Despite this, several studies have presented evidence of bacteria or bacteria-like structures within the circulation in the absence of overt disease. It should be noted, however, that Martel et al. (2017) report that the bacteria-like particles often described following a range of imaging techniques represent non-living membrane vesicles and protein aggregates derived from the blood itself. McLaughlin et al. (2002) surveyed the blood of 25 healthy donors and observed the presence of pleomorphic bacteria using dark-field microscopy, electron microscopy, polymerase chain reaction and fluorescent *in situ* hybridisation, in all samples analyzed. Further, Potgieter et al. (2015) described the presence of blood-cell associated bacteria in a range of blood preparations using electron microscopic techniques. Significantly, Damgaard et al. (2015) found viable (*culturable*) bacteria in 62% of blood donations from donors with no overt disease. This finding is plausible given that various daily activities including chewing, tooth brushing, and flossing result in the translocation of oral bacteria into the bloodstream (Forner et al., 2006; Lockhart et al., 2008; Parahitiyawa et al., 2009; Horliana et al., 2014), however, one would expect such organisms to be rapidly targeted and removed with by the immune system in healthy individuals. Kell and Pretorius (2015) provide a detailed hypothesis for the existence of the blood microbiome (Potgieter et al., 2015) and suggest that it is likely composed of organisms (or parts thereof) that translocate to the circulation from their usual place of habitation (classical niches such as the gastrointestinal tract, oral cavity, skin, vagina) – a process termed atopobiosis. They further describe how this could occur via well-described physiological processes including dendritic cells processes, via micro-fold cells, and in disease, via dysfunctional epithelial junctions. This explanation is supported by studies demonstrating a correlation between gut microbiota dysbiosis and altered microbial signatures detected in the blood (Ono et al., 2005; Sato et al., 2014; Lelouvier et al., 2016), suggesting that the observed disease-associated blood microbiota is a consequence of increased bacterial translocation across the gut barrier. Furthermore, characterization of the microbial populations in the coronary artery tissues by Lehtiniemi et al. (2005) identified known members of the oral microbiota, suggesting that bacteria had translocated from the oral cavities into the bloodstream, potentially as a result of damage caused by tooth brushing or by leakage across the mucosal surfaces.

Various disease states are associated with blood microbiome dysbiosis (Amar et al., 2013; Sato et al., 2014; Lelouvier et al., 2016; Mangul et al., 2016; Ling et al., 2017), and these changes are likely reflective of dysbiosis at a distant site(s) with well-characterized microbial communities, and the result of subsequent translocation. Limited evidence also suggests that these changes may be disease-specific; Alzheimer’s disease for example, has been associated with the detection of mostly coccus microbes, whilst Parkinson’s disease has been associated with both coccus and bacillus bacteria (Potgieter et al., 2015). Such changes are of significant scientific and medical interest as they offer opportunities for biomarker and therapeutic target development (Lelouvier et al., 2016; Ling et al., 2017).

Due to the long-held belief that the bloodstream of healthy individuals is sterile and since the blood is an unfavorable compartment for the microbes due to its bacteriostatic and bactericidal components (Mandal et al., 2016; Markova, 2017), it is of principal significance to understand *whether* and *how* bacteria may persist in it. Accidental contamination during the collection of blood and or during downstream experimental procedures has been proposed as an alternative explanation for the existence of the blood microbiome *per se*. We support this explanation for the detection of *viable* bacteria within the bloodstream of healthy individuals, however, suggest that this phenomenon does not adequately explain the existence of the blood microbiome (*the presence of bacteria-derived nucleic acids*) when one considers the number of studies that demonstrate significant and apparently disease-specific differences in the composition of the blood microbiome. Moreover, examination of the bacterial taxa reported in these studies reveal similar blood microbiota compositions across the different studies, whereby *Proteobacteria* dominate (relative abundance values typically ranging from 85 to 90%), and *Firmicutes*, *Actinobacteria*, and *Bacteroidetes* present to a lesser extent (Amar et al., 2013; Lelouvier et al., 2016; Païssé et al., 2016; Olde Loohuis et al., 2018). This suggests the existence of a core blood microbiome profile that persists independent of study environment or analytical methodology.

**FIGURE 1:**
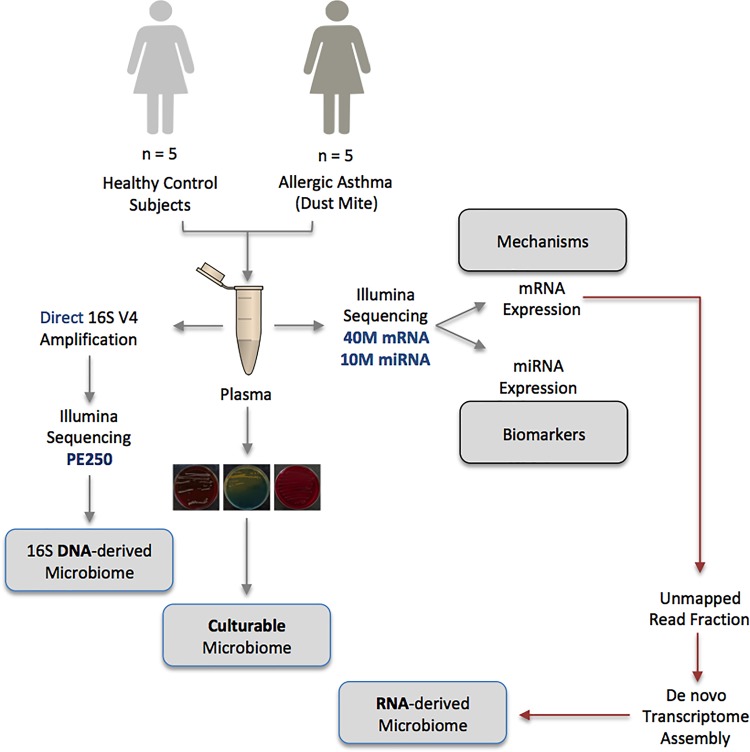
Schematic representation of the multiple-method circulating microbiome characterization approach implemented herein. NB: Biomarker and mechanistic data are not included within the scope of this publication and appear elsewhere.

Using a range of complementary molecular and classical molecular biology techniques, the human circulating microbiome was characterized in unparalleled detail; at the DNA level, the 16S rRNA gene was amplified and sequenced, at the RNA level, almost 500,000,000 unmapped mRNA reads were assembled and mapped to known taxa, and finally, viability data was generated using classical aerobic and anaerobic microbial culture experiments. The experimental approach is detailed in Figure 1.

## Materials and Methods

### Donor Samples

Atopic asthmatic individuals (*n* = 5) with physician-diagnosed house dust mite allergy, and gender and age-matched healthy control subjects (*n* = 5) were recruited to the study via SeraLabs Limited in accordance with the following criteria (Table 1). Whole blood was drawn, following alcohol cleansing of the skin surface, into EDTA containing tubes and stored on ice prior to centrifugation at 1000 ×*g* to obtain the plasma component. All samples were analyzed anonymously, and the authors obtained ethical approval (Keele University ERP3) and written informed consent to utilize the samples for the research reported herein.

**Table 1:** Donor population characteristics.

Patient Criteria
• Have a BMI < 30
• Be non-smokers
• Were diagnosed with atopic asthma during childhood
• Have severe/ poorly controlled symptoms
• Are not on current oral steroid treatment
• Must be allergic to house dust mite
• Must not have diabetes, COPD, or hypertension


### DNA-Level: Meta-Taxonomic Characterization

The circulating microbiome was investigated at the DNA level by amplification and sequencing of the bacterial 16S ribosomal gene using oligonucleotide primers that target variable region 4 (Kozich et al., 2013) (Table 2). Direct amplification of the V4 region was performed using the Phusion Blood Direct kit (Thermo Fisher Scientific) alongside a negative control reaction (in which blood was replaced with molecular biology grade water) that underwent the complete experimental procedure. Amplification was performed in triplicate as 20 μl reactions containing; 1.0 μl plasma, 10 μl 2X Phusion blood PCR buffer, 0.4 μl Phusion Blood II DNA polymerase, 1.0 μl of each forward and reverse oligonucleotide primer (10 μM), and 6.6 μl of UV-treated molecular biology grade water. Cycling parameters were as follows; an initial 5 min denaturation step at 98°C followed by 33 cycles of; denaturation (1 s at 98°C), annealing (5 s at 55°C), and extension (15 s at 72°C), and a final extension at 72°C for 7 min.

**Table 2:** Oligonucleotide primer sequences.

Primer Name	Sequence (5′–3′)	Length
V4_F	GTGCCAGCMGCCGCGGTAA	19
V4_R	GGACTACHVGGGTWTCTAAT	20
V4_XT_F	TCGTCGGCAGCGTCAGATGTGTATAAGAGACAGGTGCCAGCMGCCGCGGTAA	52
V4_XT_R	GTCTCGTGGGCTCGGAGATGTGTATAAGAGACAGGGACTACHVGGGTWTCTAAT	54


Amplicons resulting from triplicate reactions were combined and purified using the MinElute PCR purification kit (Qiagen) prior to a further 7 cycles of PCR using AccuPrime *Pfx* SuperMix and a pair of V4 oligonucleotide primers we developed to incorporate the Illumina XT adapter in preparation for sequencing (Table 2). Cycling parameters were as follows; initial denaturation for 2 min at 95°C followed by 7 cycles of; denaturation (15 s at 95°C), annealing (30 s at 55°C), and extension (25 s at 68°C), and a final extension at 68°C for 10 min. PCR products were purified using the AMPure XP magnetic beads (Agencourt) at a ratio of 0.8 beads to sample (*v*/*v*), eluted in 20 μl molecular biology grade water, and quantified using the Qubit 3.0 high-sensitivity DNA kit. Amplicons were barcoded using the Nextera DNA library kit, multiplexed, and sequenced using the Ilumina MiSeq system with a 250 bp paired-end read metric. Bioinformatic analysis was performed using QIIME (Caporaso et al., 2010) implemented as part of the Nephele 16S paired-end QIIME pipeline using closed reference clustering against the SILVA database (Quast et al., 2013) at a sequence identity of 99%. All other parameters remained as default.

### RNA-Level: *De novo* Assembly of Unmapped RNA Reads

For the purposes of increasing our understanding of the molecular processes that are deregulated in patients with atopic asthma, we previously performed whole transcriptome sequencing on RNA extracted from each donor plasma sample (data currently unpublished). Here, we hypothesized that the non-mapping (likely non-human) reads that often result from such analyses would represent microbial community members that were present in the blood at the time of RNA extraction. To this end, RNA reads were mapped to the *Homo sapiens* genome version hg38 using Tophat with default parameters (Trapnell et al., 2012). Reads failing to map to hg38 were identified from the resulting BAM file and reads, in fastq format, extracted using bedtools bamtofastq. In order to streamline our strategy, a single de novo transcriptome was produced by concatenating all of the unmapped reads produced from the entire study, and assembling these using Trinity (Haas et al., 2013) to form features representative of candidate non-human genes. To increase computational efficiency, the total read population was subsampled to a depth of 1, 10, 25, and 100 M, and the number of reads that these features explained computed by building a bowtie2 index out of each resulting transcriptome, and mapping the unmapped read population from each sample back to it. The transcriptome assembly with the highest mapping rate was used for further analysis as follows: (1) Abundance estimation – the transcriptome was indexed for bowtie2 and the number of reads mapping to each feature determined for each of our donor samples using RSEM (Li and Dewey, 2011). (2) A matrix of expression values was produced using the abundance_estimates_to_matrix.pl script packaged with Trinity (Grabherr et al., 2011). (3) Statistical analysis – a differential expression analysis was conducted to identify candidate non-human genes that were significantly differentially expressed between the disease and disease-free donors using edgeR (Robinson et al., 2010). (4) Identification – the taxonomic identity of each assembled feature was determined using Kraken (Wood and Salzberg, 2014) and visualized using Pavian (Breitwieser and Salzberg, 2016).

### Classical Culture: Bacterial Viability

Classical microbiological culture, using a range of substrates, was carried out to determine whether the human plasma samples contained any viable bacterial cells, i.e., those capable of proliferation. For each sample, 250 μl plasma was inoculated into 9 ml of brain heart infusion broth and incubated for 5 days at 37°C. For each culture a negative control was generated, whereby 9 ml of brain heart infusion broth was inoculated with 250 μl of ultra-violet sterilized distilled water and incubated for 5 days at 37°C. The inoculated broth was plated onto agar plates (Columbia agar + 5% horse blood; CLED medium; A.R.I.A + horse blood) and incubated under either aerobic (Columbia blood agar; CLED medium) or anaerobic (A.R.I.A agar) conditions at 37°C for a minimum of three days. Bacterial growth was evaluated by sight, and a selection of individual colonies from each plate was selected for identification by total 16S gene amplification and Sanger sequencing.

## Results and Discussion

### Donor Characteristics

This study utilized samples originally sourced for the molecular characterization of asthma pathogenesis^1^. The donor population were all female (due to the characteristics of the clinical donor population at the time of collection), and all “never smokers.” The asthmatic population were 39.6 years in age (range 19–52) with a mean body mass index of 24.4 (range 21.5–27.8) and all had physician-diagnosed atopic asthma resulting from house dust mite allergy. The control population were 39.4 years in age (range 23–49) with a mean body mass index of 24.3 (range 21.0–26.4) and were disease-free. There were no statistically significant differences in age (*P* = 0.98) or BMI (*P* = 0.93) between the two groups (Table 3).

**Table 3:** Donor population characteristics.

Donor	Age (years)	BMI (kg/m^2^)	Smoking History	Diagnosis
BRH1017873	50–60	27.8	Never	Atopic Asthma, HDM
BRH1017874	30–40	27.3	Never	Atopic Asthma, HDM
BRH1017875	40–50	23.3	Never	Atopic Asthma, HDM
BRH1017876	10–20	21.5	Never	Atopic Asthma, HDM
BRH1017877	40–50	22.3	Never	Atopic Asthma, HDM
BRH1017878	40–50	26.4	Never	Healthy
BRH1017879	20–30	21	Never	Healthy
BRH1017880	40–50	26.4	Never	Healthy
BRH1017881	40–50	24.9	Never	Healthy
BRH1017882	30–40	22.7	Never	Healthy


### DNA-Level Circulating Microbiome – Community Structure

The presence of bacterial DNA within the blood of our study cohort was evaluated by amplification and sequencing of the 16S RNA gene variable region 4. A negative experimental control sample mirrored our study samples through the entire experimental procedure downstream of venepuncture. This involved using ultra-violet treated molecular biology-grade water in replacement of human blood during PCR amplification of the 16S rRNA V4 region. The negative control PCR product was then submitted to all downstream applications that the human blood underwent. This included bead-based purification of the 16S rRNA V4 amplicons, agarose gel electrophoresis, XT-tagging, library preparation and sequencing of the 16S rRNA V4 amplicons.

**FIGURE 2:**
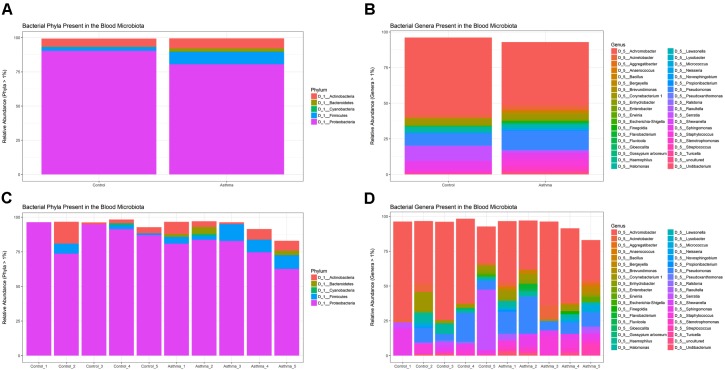
Relative abundance of the most abundant taxa (>1%) as determined by amplification and sequencing of the 16S rRNA gene variable region 4. Data are mean abundance expressed as a percentage of the total bacterial sequence count. **(A)** Phylum-level data grouped by condition, **(B)** Genus-level data grouped by condition, **(C)** Phylum-level individual sample data, and **(D)** Genus-level individual sample data.

Using QIIME (Caporaso et al., 2010) implemented as part of Nephele, a total of 243, 853 sequencing reads from the amplified V4 region passed quality assessment (mean = 24,385 reads per sample; range = 10,742–35,701 reads). The results of our taxonomic classification are shown in (Figures 2A–D) at the phylum and genus levels.

Our negative control reaction generated a small number of reads that were identified as the following genera; *Halomonas (6 reads)*, *Corynebacterium (64)*, *Staphylococcus (24)*, *Ralstonia (1726)*, *Stenotrophomonas (460)*, *Pseudomonas (276)*, *Escherichia*-*Shigella (2420)*, and *Ruminococcus* (405), but was overwhelmingly composed of reads mapping to the genus *Serratia (18000)*. These genera have been reported previously as contaminants of next generation sequencing experiments (Laurence et al., 2014; Salter et al., 2014) but importantly here, were either distinct from the taxa identified within our samples, or present at much lower levels.

At the phylum level, the blood microbiome was predominated by *Proteobacteria* (88% of all bacterial DNA in the control population, and 80.9% in the asthmatic population) followed by *Actinobacteria* (control = 7.8%, asthmatic = 7.1%), *Firmicutes* (control = 3.5%, asthmatic = 9.2%) and *Bacteroidetes* (control = 0.1%, asthmatic = 2.2%). These findings mirror previous studies (Amar et al., 2013; Lelouvier et al., 2016; Païssé et al., 2016; Olde Loohuis et al., 2018) and further support the notion of a core blood microbiome predominated by four key phyla.

At the genus level, our blood samples were predominated by the genus *Achromobacter* (Gyarmati et al., 2016) which accounted for 51.1 and 45.3% of the total bacterial DNA detected in the control and asthma donors, respectively. To a lesser extent, the blood samples also comprised members of the *Pseudomonas* (12.8%, 7.5%) (Dinakaran et al., 2014; Damgaard et al., 2015; Païssé et al., 2016), *Serratia^∗^* (0.9%, 11.6%) (Moriyama et al., 2008), *Sphingomonas* (3.8%, 5.1%) (Lelouvier et al., 2016; Païssé et al., 2016), *Staphylococcus* (5.5%, 2.8%) (Such et al., 2002; Marques da Silva et al., 2003; Damgaard et al., 2015; Païssé et al., 2016), *Corynebacterium* (3.2%, 5.5%) (Dinakaran et al., 2014; Païssé et al., 2016), and *Acinetobacter* (3.7%, 2.8%) (Païssé et al., 2016) genera. The genus *Serratia* was excluded from further analysis as the study samples presented with less reads than did the corresponding negative control reaction and thus it was considered a contaminant.

Whilst all genera found have been previously described in the blood (see references), the predominance of *Achromobacter*, which is not classically associated with the blood microbiome, warrants further consideration. Indeed, *Achromobacter* has been detected abundantly in the lower respiratory tract of healthy mice (Singh et al., 2017), humans (HPM airway dataset), and in various respiratory conditions (Segal et al., 2014; Hogan et al., 2016). Furthermore, no *Achromobacter* was detected in our experimental control reactions suggesting that its presence is not the result of experimental contamination.

**FIGURE 3:**
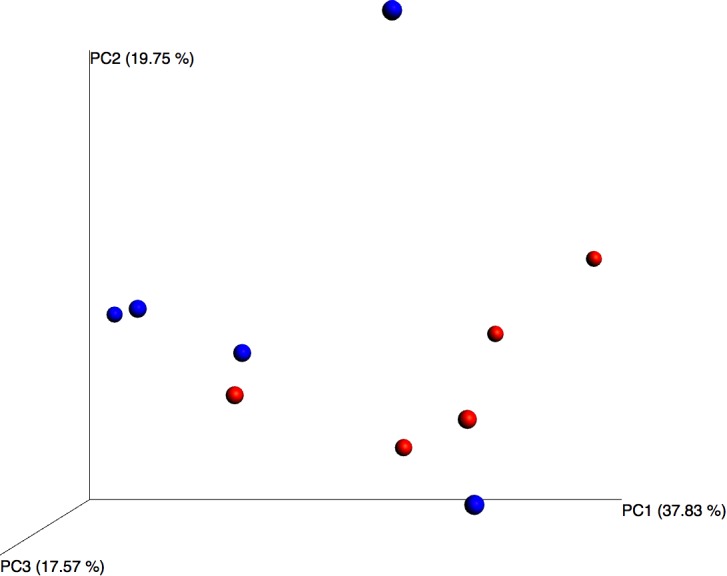
Principal coordinates analysis of weighted unifrac distances for control (blue) and asthmatic (red) blood microbiome profiles. Each dot represents an individual sample, and the microbiome of samples that appear more closely together are more similar.

Although differential composition of the blood microbiome in response to pathology was not the main focus of this study, we performed principal coordinates analysis and despite the relatively small sample set, this revealed good separation between the two treatment groups based upon their microbiome profile, suggesting that the blood microbiome community was altered in the asthmatic subjects (Figure 3).

### DNA-Level Circulating Microbiome – Likely Origins

**FIGURE 4:**
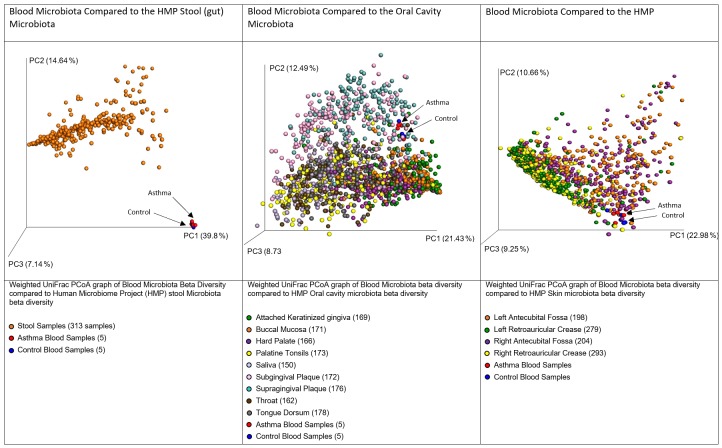
Principal coordinates analysis of weighted unifrac distances between variable region 4 16S sequencing data from our donors and the Human Microbiome Project Gut, Oral Cavity, and Skin data. Each dot represents an individual sample, and the microbiome of samples that appear more closely together are more similar. In each case, our control donor samples appear in blue, and our asthmatic donor samples appear in red. Further sample details are provided beneath each figure, and the number of datasets representing each anatomic location is provided in brackets.

In accordance with our hypothesis that the blood microbiome exists as a consequence of bacterial translocation from other microbiome niches within the body, we compared the data generated herein with gastrointestinal tract, oral cavity, and skin data made available by the Human Microbiome Project (HMP). In each case, the HMP data and our own were combined, weighted UniFrac distances calculated, and a principal coordinates analysis performed. Figure 4 demonstrates that the blood microbiome of our control and asthmatic donors clustered more closely in PCoA space with the oral cavity and skin HMP data, than it did with the gastrointestinal tract HMP data. This suggests that the blood microbiome community is perhaps more likely to result from the translocation of organisms from the oral cavity and skin, than from organisms that colonize the gastrointestinal tract.

Various daily activities including chewing, tooth brushing and flossing have been shown to result in the translocation of bacteria from the oral cavity into the bloodstream (Forner et al., 2006; Lockhart et al., 2008; Parahitiyawa et al., 2009; Horliana et al., 2014). Further, the skin has a distinct microbial community and is susceptible to injury, and thus represents a large surface area through which such organisms may translocate to the bloodstream. It is important here to consider sources of contamination; venepuncture, the process through which the majority of blood samples are obtained, is recognized as a cause of transient bacteraemia (Depcik-Smith et al., 2001), and despite the use of preventative measures such as alcohol cleansing of the skin prior to breaking the surface, there remains the possibility that organisms entered the sample from the skin via this route.

One important, but often overlooked limitation of DNA-based microbiome characterization is that DNA persists post-mortem, even in the presence of harsh environmental conditions. From such analyses it is therefore impossible to confirm whether an organism is present and viable, is present but non-viable, or whether the organism has since left the environment in question yet it’s DNA persists.

### RNA-Level Circulating Microbiome – Community Structure

**FIGURE 5:**
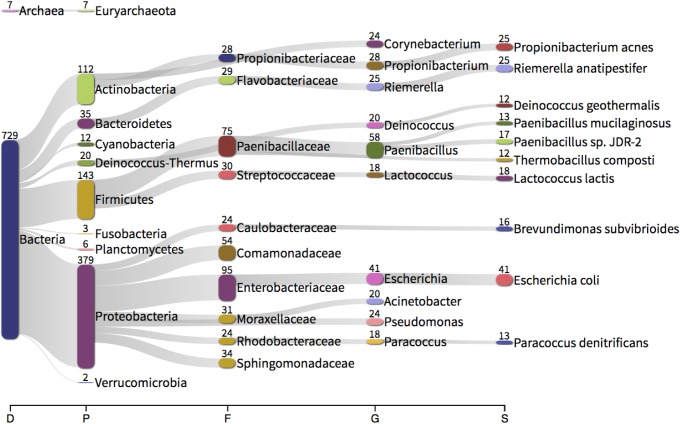
Taxonomic classification of each feature assembled from unmapped RNA sequencing reads using Trinity and identified using Kraken. The numbers present by each taxonomic classification refer to the number of features classified as such (e.g., 379 assembled features were identified as Proteobacteria). D – domain, P – phylum, F – family, G – genus, S – species.

We hypothesize that some of the non-mapping (likely non-human) reads that often result from whole transcriptome analyses (RNA-seq) represent microbial community members that were present (or parts thereof) in the blood at the time of RNA extraction. Furthermore, given the unstable nature of extracellular circulatory RNA (Tsui et al., 2002) in addition to the presence of circulatory ribonucleases that actively degrade RNA (Segal et al., 2014), we suggest that the detection of bacterial RNA goes further toward confirming the recent presence of these microbes within the blood when compared with DNA-based approaches. From our previous studies of the circulating transcriptome of our donor community, a total of 439,448,931 paired RNA reads failed to map to the human genome. These reads were used for the following analyses as randomly subsampled populations of 1, 10, 25, and 100 million read pairs. Mapping the total read population back to the subsampled populations allowed us to assess how well each subsampled population approximated the starting (entire, ∼ 440 M read) population. Data revealed only marginal improvements in whole community representation as the subsampled population increased (1, 10, 25, and 100 M represented 65.05, 66.05, 66.81, and 64.24% of the total read population). A subsampled population of 25 million reads (25 M) provided an acceptable balance between read representation and computational efficiency and was therefore used for transcriptome assembly. The transcriptome comprised 2050 candidate “non-human genes” with a mean GC content of 53%. Ten-percent of these genes were greater than 517 bp, and over half were at least 263 bp. Taxonomic identification of each assembled feature was determined using Kraken (Wood and Salzberg, 2014) and this revealed that 729 of the 2050 features were of bacterial origin and seven features were from archaea [pertaining to the taxa *Thermoplasmata*, which has been previously associated with the human microbiome (Lurie-Weinberger and Gophna, 2015)] (Figure 5). Although we identified 13 features of apparent viral origin, we did not consider these in any further detail given they appeared to pertain to the Moloney murine leukemia virus, a commonly utilized reverse transcriptase used in molecular procedures. It should be noted that the Kraken database does not include fungi, and therefore this kingdom was not represented within our data.

At the phylum level, the whole transcriptome data was predominated by assembled *Proteobacteria* sequences (379 sequences, 52%), followed by *Firmicutes* (143, 19.8%), *Actinobacteria* (112, 15.5%) and *Bacteroidetes* (35, 4.8%). In considering the total number of reads mapping to each feature, 379.4 M reads mapped to bacterial features (out of a total of 395 M reads). Of those reads mapping to bacterial-derived sequences, *Proteobacteria* (74.9%, 47.0%; Control, Asthma) and *Firmicutes* (19.5%, 48.0%) predominated with, *Actinobacteria* (0.01%, 0.04%) and *Bacteroidetes* (0.05%, 0.008%) present to a much lesser extent. These findings support our DNA-level phylum data and mirror previous studies (Amar et al., 2013; Lelouvier et al., 2016; Païssé et al., 2016; Olde Loohuis et al., 2018).

At the genus level, the whole transcriptome data was predominated by the genera *Paenibacillus* (17.8%, 44.6%; Control, Asthma reads) (Sáez-Nieto et al., 2017), *Escherichia* (11.8%, 13.1%) (Such et al., 2002; Francés et al., 2004; Dinakaran et al., 2014; Païssé et al., 2016), *Acinetobacter* (0.7%, 0.4%) (Païssé et al., 2016), *Pseudomonas* (0.8%, 0.1%) (Bhatt et al., 2014; Dinakaran et al., 2014; Damgaard et al., 2015; Païssé et al., 2016) and *Propionibacterium* (0.5%, 0.2%) (Marques da Silva et al., 2003; Bhatt et al., 2014; Dinakaran et al., 2014; Damgaard et al., 2015). With the exception of *Paenibacillus*, all genera detected via *de novo* transcriptome assembly were also present in our DNA-level data and all genera found have been previously described in the blood (see references). The fact that *Paenibacillus* was present within our RNA-level analyses yet absent from our DNA-level data led us to consider whether it could have been introduced as a contaminant during the RNA extraction, library preparation or sequencing procedures. Given that negative control reactions are not routinely conducted for RNA-seq type applications, we were unable to experimentally confirm this. However, we did identify from the literature that this genus has been reported as a common reagent and laboratory contaminant, albeit at the DNA level (Salter et al., 2014). Nevertheless there appeared to be little consistency in its presence within our sample set (mean % of reads 39.5 ± 40.3%) and we noted a difference in abundance between our experimental groups despite all preparation procedures being the same. The exact source of this RNA thus remains open to speculation.

### Classical Culture – Presence of Viable Organisms

The presence of viable, proliferating bacteria in the blood was assessed using growth culture assays as previously described. Bacterial cultures were positive for 80% of blood samples assayed (8 out of 10 samples; 4 control blood samples and 4 asthma blood samples), whilst all negative control plates had no growth as expected. Negative cultures were repeated on two further occasions to confirm this status. These results are relatively consistent with previous studies, whereby 2–100% of blood samples were positive for bacterial growth (Wilson et al., 1975; Domingue and Schlegel, 1977; Jiménez et al., 2005; Damgaard et al., 2015; Panaiotov, 2018). Unexpectedly, bacterial growth was observed in aerobic conditions for all culture-positive blood samples, but anaerobic growth was only observed for four of the culture-positive blood samples. This is contradictory to previous studies, where bacterial growth from blood-cultures has predominately been achieved using anaerobic conditions (Jiménez et al., 2005; Damgaard et al., 2015).

**FIGURE 6:**
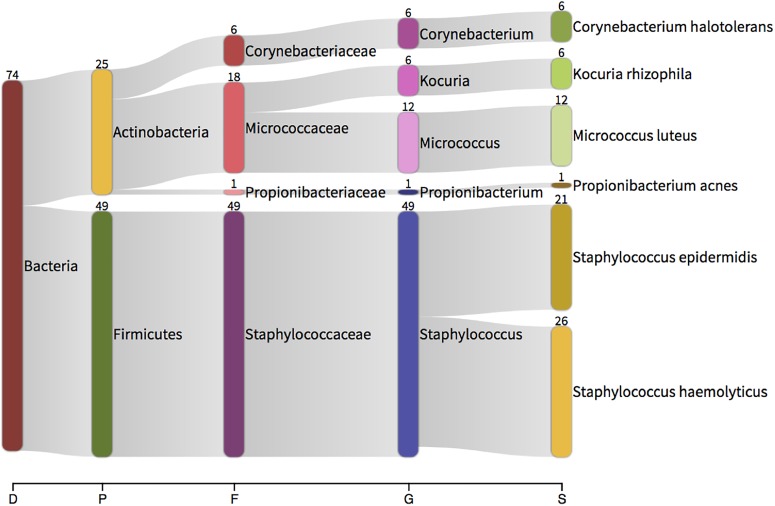
Taxonomic classification of total 16S data generated by colony PCR and Sanger sequencing. The numbers present by each taxonomic classification indicate the number of colonies that were identified with that identity. D – domain, P – phylum, F – family, G – genus, S – species.

In all instances, bacterial growth was monoculture on microscopy and thus 16S colony PCR (amplifying the entire 16S rRNA gene) and Sanger sequencing was conducted on a three independent colonies per plate for identification purposes. Bacteria were identified using Sanger sequencing followed by classification with Kraken. Bacteria isolated from the aerobic cultures included the following genera; *Staphylococcus* (49 sequences), *Micrococcus* (12), *Kocuria* (6), *Corynebacterium* (6) and *Propionibacterium* (1). Bacteria isolated from the anaerobic cultures were less variable and included members from the facultatively anaerobic *Staphylococcus* genus only (Figure 6). These genera belong to the phyla *Actinobacteria* (*Corynebacterium*, *Kocuria*, *Micrococcus*) and *Firmicutes* (*Staphylococcus*) and were all represented in our 16S DNA level data (Figure 4) and detected within our RNA data. Individual sample data is presented in Table 4. It is noteworthy that, with the exception of *Kocuria*, all bacteria identified displayed some of the highest total relative abundance scores in the 16S sequencing results; *Corynebacterium* (4.2%), *Kocuria* (0.2%), *Micrococcus* (1.30%), and *Staphylococcus* at 4.3%. Due to the long-held belief that the bloodstream of healthy individuals is sterile and since the blood is an unfavorable compartment for the microbes due to its bacteriostatic and bactericidal components; here we consider the likely source of these viable organisms. The skin microbiome is dominated by members of the genera *Corynebacterium*, *Micrococcus*, *Staphylococcus*, and *Propionibacterium*, the proportions of which vary markedly between individuals (Tett et al., 2017). Furthermore, several studies report the presence of the genus *Kocuria* on the skin of humans and other mammals (Grice et al., 2008; Cosseau et al., 2016; McIntyre et al., 2016). We therefore suggest that the organisms detected through our microbial culture experiments most likely originate from the skin. Whilst transient bacteraemia due to a breach of the skin barrier is an accepted occurrence, one would expect such organisms to be rapidly targeted and removed by the immune system.

**Table 4:** Identification of cultured organisms using 16S colony PCR and Sanger sequencing.

Growth Condition	Control Samples					Asthma Samples				
		
	BRH1017878	BRH1017879	BRH1017880	BRH1017881	BRH1017882	BRH1017874	BRH1017875	BRH1017876	BRH1017877
Aerobic Growth	Kocuria *rhizophila*	Micrococcus *luteus*	Staphylococcus *haemolyticus* Staphylococcus *epidermidis* Propionibacterium *acnes*	Staphylococcus *haemolyticus*	Culture Neg	Staphylococcus *haemolyticus*	Culture Neg	Corynebacterium *halotolerans*	Staphylococcus *epidermidis*
Anaerobic Growth	Culture Neg	Culture Neg	Staphylococcus epidermidis	Staphylococcus hominis	Culture Neg	Culture Neg	Culture Neg	Culture Neg	Staphylococcus epidermidis


*Culture Neg denotes no bacterial growth on the substrates used.*

We therefore suggest that the viable organisms detected through classical microbial culture analysis are the result of venepuncture contamination whereby organisms from the skin are drawn into the vacutainer, contaminating the sample. An alternative hypothesis suggests that these bacteria were present in the blood in a dormant state (i.e., not contaminants), and were somehow revived following pre-growth in brain heart infusion broth prior to plating [see Kell and Pretorius, 2015 for a detailed description of this hypothesis (Potgieter et al., 2015)], however, this hypothesis is still under intense investigation.

## Conclusion

This study utilized a range of molecular and classical microbiology approaches to characterize the human blood microbiome in unparalleled detail. Our DNA and RNA-based studies revealed a diverse community of bacteria, the main members of which having been described in a range of other studies and therefore providing further evidence of a core blood microbiome. Although disease associated changes in the blood microbiome were not the focus of this study, the fact we identified such changes is encouraging and supports efforts to identify circulating microbiome signatures indicative of disease.

Whilst we attribute the finding of viable organisms in our plasma samples to venepuncture-associated contamination of the blood sample (and make recommendations to avoid this in future) and or the phenomena of dormancy, the presence of these viable organisms does not undermine our exciting molecular data that reports an abundance of bacteria-associated DNA and RNA within the blood, likely present due to translocation from classical microbiome niches (such as the gut, oral cavity and skin), and with the clear potential to be developed as a biomarker of microbiome status at distant anatomical sites.

On reflecting upon our experimental approach, we make the following recommendations for future studies:

(1) Significant attention should be paid to blood collection procedures as any contamination occurring at this stage impacts upon all downstream procedures. In addition to alcohol cleansing of the skin (as performed in this study), we recommend that the first volume drawn is diverted to a secondary tube, and analyzed separately. This will allow investigation of the contribution that venepuncture-associated contamination makes, and allow robust analysis of the dormancy hypothesis.
(2) The inclusion of negative control reactions that are subject to the whole range of experimental procedures, including library preparation and sequencing, is absolutely essential.
(3) Where possible, RNA-seq studies used for unmapped read assembly should include negative control samples that are subject to all of the experimental procedures alongside the study samples. This will allow an appraisal of how significant reagent/laboratory procedure contamination is in these studies. Often the use of RNA-seq derived unmapped reads for microbiome characterization is a “secondary use,” and this is simply not possible.
(4) Seminal studies are still required to satisfactorily investigate the phenomenon of bacterial translocation from well-characterized microbiome niches to the blood.

## Data Availability

The sequencing data utilized in this project can be found in the Sequence Read Archive, NIH, under the identifier SUB4654957.

## Ethics Statement

This study was carried out in accordance with the recommendations of Keele Ethical Review Panel 3 (ERP3), Keele University with written informed consent from all subjects obtained by SeraLab limited who supplied the blood samples used herein. All subjects gave written informed consent in accordance with the Declaration of Helsinki.

## Author Contributions

DT conceived the novel unmapped read approach to microbiome analysis, carried out the bioinformatic method development, and prepared the original manuscript. DT, ML, and TG designed the original study who generated the RNA sequencing data. EW conducted the 16S work, classical microbiology and assisted in the interpretation of the findings. RH managed the classical microbiology. DT, ML, EW, RH, and TG approved the final manuscript.

## Conflict of Interest Statement

The authors declare that the research was conducted in the absence of any commercial or financial relationships that could be construed as a potential conflict of interest.

## Footnotes

1 https://doi.org/10.1101/446427
